# *Mycobacterium fortuitum*-induced ER-Mitochondrial calcium dynamics promotes calpain/caspase-12/caspase-9 mediated apoptosis in fish macrophages

**DOI:** 10.1038/s41420-018-0034-9

**Published:** 2018-02-20

**Authors:** Debika Datta, Preeti Khatri, Ambika Singh, Dhira Rani Saha, Gaurav Verma, Rajagopal Raman, Shibnath Mazumder

**Affiliations:** 1 Department of Zoology, Immunobiology Laboratory, University of Delhi, Delhi, India; 2 Department of Zoology, Gut Biology Laboratory, University of Delhi, Delhi, India; 3 Microscopy Laboratory, National Institute of Cholera and Enteric Diseases, Kolkata, India; 4 Gene Regulation Laboratory, School of Biotechnology, Jawaharlal Nehru University, New Delhi, India

## Abstract

*Mycobacterium fortuitum* is a natural fish pathogen. It induces apoptosis in headkidney macrophages (HKM) of catfish, *Clarias* sp though the mechanism remains largely unknown. We observed *M. fortuitum* triggers calcium (Ca^2+^) insult in the sub-cellular compartments which elicits pro-apototic ER-stress factor CHOP. Alleviating ER-stress inhibited CHOP and attenuated HKM apoptosis implicating ER-stress in the pathogenesis of *M. fortuitum*. ER-stress promoted calpain activation and silencing the protease inhibited caspase-12 activation. The study documents the primal role of calpain/caspase-12 axis on caspase-9 activation in *M. fortuitum*-pathogenesis. Mobilization of Ca^2+^ from ER to mitochondria led to increased mitochondrial Ca^2+^ (Ca^2+^)_m_ load,_,_ mitochondrial permeability transition (MPT) pore opening, altered mitochondrial membrane potential (ΔΨm) and cytochrome *c* release eventually activating the caspase-9/-3 cascade. Ultra-structural studies revealed close apposition of ER and mitochondria and pre-treatment with (Ca^2+^)_m_-uniporter (MUP) blocker ruthenium red, reduced Ca^2+^ overload suggesting (Ca^2+^)_m_ fluxes are MUP-driven and the ER-mitochondria tethering orchestrates the process. This is the first report implicating role of sub-cellular Ca^2+^ in the pathogenesis of *M. fortuitum*. We summarize, the dynamics of Ca^2+^ in sub-cellular compartments incites ER-stress and mitochondrial dysfunction, leading to activation of pro-apoptotic calpain/caspase-12/caspase-9 axis in *M. fortuitum*-infected HKM.

## Introduction

*M. fortuitum* is a rapidly growing, atypical, non-tubercular mycobacteria affecting wide range of animals including humans^1–3^. In fish, it is one of the etiologic agents causing piscine-tuberculosis or mycobacteriosis, a fatal disease characterized by the development of gray-white nodular structures and presence of single or multiple granulomatous lesions on several parenchymal organs ^1^. Despite its diverse host trophism and zoonotic importance, our knowledge on pathogenic mechanisms and virulence factors expressed by *M. fortuitum* is incomplete.

Alterations in cytosolic calcium (Ca^2+^)_c_ levels play crucial role in microbial pathogenesis and disease outcome with reports suggesting pro-and anti-apoptotic roles of Ca^2+^ on mycobacteria-infected macrophages^4, 5^. Once Ca^2+^ is mobilized, it either interacts with various Ca^2+^**-**binding proteins or gets sequestered into the ER^6^. Calcium influx or depletion from the ER induces ER-stress^6, 7^. The ability to mount ER-stress response is critical for cell survival, but chronic or unresolved ER stress can lead to expression of pro-apoptotic C/EBP homologous protein (CHOP)^8^. Though prolonged ER-stress has been linked to mycobacterial pathogenesis^9–14^, it has not been reported in *M. fortuitum*.

To mitigate stress, the ER releases Ca^2+^ ((Ca^2+^)_ER_) through ER-membrane resident inositol-1,4,5-trisphosphate receptors (IP_3_R) and ryanodine receptors (RYRs)^15^. The (Ca^2+^)_ER_ is either pumped out of the cell through specific channels or taken up by mitochondria through specific uniport transporter like M1CU1 and VDAc, the latter being facilitated by the known proximity between the two organelles^16, 17^.

Calcium overload to mitochondria leads to mitochondrial structure-function alterations eventually releasing the pro-apoptotic cytochrome *c* to the cytosol^17^. Activation of caspases, a family of cysteine-dependent aspartate-directed proteases, is central to apoptosis and caspase-12 appears to be the prime caspase involved in ER-stress induced apoptosis^18^. Calpains are Ca^2+^-activated non-lysosomal cysteine proteases which exist in two isoforms, calpain-1 and calpain-2^19^. Each calpain consists of an 80 kDa catalytic subunit and a common 28 kDa subunit^19^. The role for calpain in promoting mycobacteria-induced apoptosis is still under investigation^10, 11, 20^. Several reports suggested the role of calpains in the activation of caspase-12^21, 22^ implicating the plurality of Ca^2+^ involvement in apoptosis.

The fish immune system is well-developed and comprised of both innate and adaptive immunity. However, unlike other vertebrates, the head kidney (HK) represents the main immunocompetent organ and HKM are important constituents of fish innate immunity^23^. We recently demonstrated the role of caspase-8 in *M. fortuitum* infection induced HKM apoptosis^24^. However, the interaction of caspase-12 and caspase-9 is not reported in *M. fortuitum* pathogenesis. In the present study we investigated the the role of caspase-12 and caspase-9 in *M. fortuitum* pathogenesis. Our results for the first time implicate Ca^2+^ dynamics between ER and mitochondria important for *M. fortuitum* induced apoptosis. We suggest that ER-stress espouses apoptosis of *M. fortuitum*-infected HKM and activation of calpain/caspase-12/caspase-9 axis crucial for initiating the apoptotic cascade.

## Results

### *M. fortuitum*-induced intracellular Ca^2+^ imbalance lead to CHOP- mediated HKM apoptosis

Previously, we reported that the imbalance in (Ca^2+^)_c_ triggers apoptosis in *M. fortuitum-*infected fish macrophages^24^. Here, we studied the dynamics of (Ca^2+^)_c_ in the two sub-cellular compartments, ER and mitochondria.

**Fig. 1:**
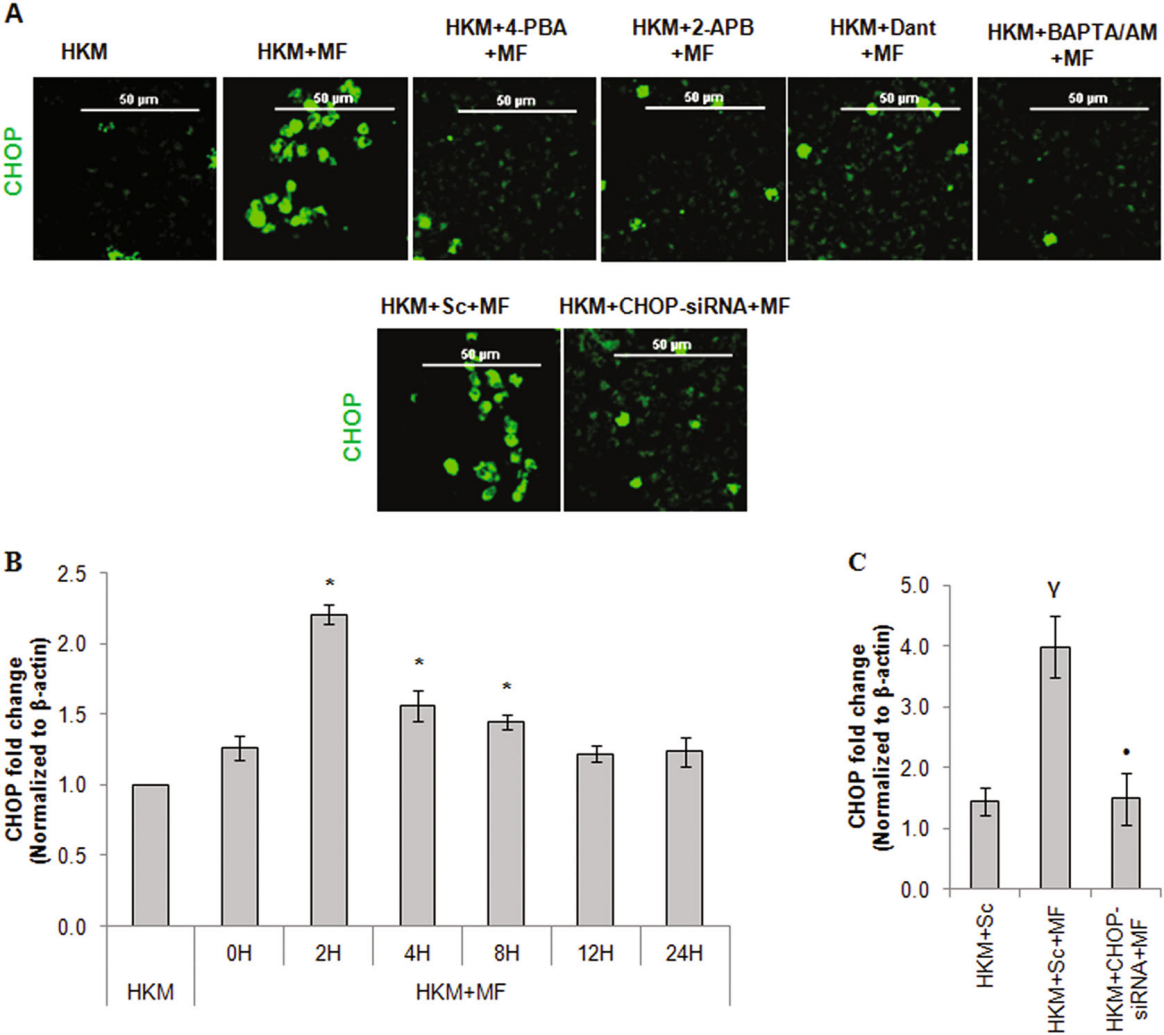
*M. fortuitum* induces CHOP- mediated HKM apoptosis. **a** HKM pre-treated or transfected with indicated inhibitors or siRNAs respectively prior to the infection with *M. fortuitum* and the CHOP protein expression was studied by confocal microscope using FITC-conjugated secondary antibody. The images are representative of three independent experiments and observed under confocal microscope ( × 40). **b** HKM were infected with *M. fortuitum* and CHOP mRNA expression was quantified by qPCR at indicated time p.i. **c** HKM were transfected with CHOP-siRNA or scrambled siRNA prior to infection with *M. fortuitum* and CHOP mRNA expression was quantified. Vertical bars represent mean ± SE (*n* = 3).*P < 0.05, compared to HKM; ^**γ**^*P* < 0.05, compared to HKM + Sc; ^**•**^*P* < 0.05, compared to HKM + MF + Sc. HKM, control headkidney macrophage; HKM + MF, HKM infected with *M. fortuitum*; HKM + Sc + MF, HKM transfected with scrambled siRNA followed by *M. fortuitum* infection; HKM + CHOP-siRNA + MF, HKM transfected with CHOP-siRNA infected with *M. fortuitum*; HKM + 4-PBA + MF, HKM + 2-APB + MF, HKM + Dant + MF, HKM + BAPTA/AM + MF, HKM pre-treated with 4-PBA, 2-APB, Dant, BAPTA/AM respectively followed by *M. fortuitum* infection

ER is the main storehouse of intracellular Ca^2+^ and under stressed condition (Ca^2+^)_ER_ is released through IP_3_R and RYRs located on the ER-membrane. CHOP is a marker for ER-stress^6, 7^ and our preliminary results suggested significant CHOP mRNA expression at 2 h (Fig. 1b) and protein at 24 h (data not shown) in *M. fortuitum-*infected HKM. The HKM were pre-treated with 2-APB and Dant, specific inhibitors for IP_3_R and RYR respectively^25^, infected with *M. fortuitum* and the changes in CHOP expression and apoptosis studied at 24 h p.i. We observed decreased expression of CHOP (Fig. 1a) and HKM apoptosis (Figure S1) which suggested positive co-relation between (Ca^2+^)_ER_ depletion and CHOP expression in *M. fortuitum* infected HKM. In the same line, we observed declined expression of CHOP in presence of intracellular Ca^2+^ chealator BAPTA/AM (Fig. 1a).

Pre-treatment of HKM with general ER-stress inhibitor 4-PBA down-regulated CHOP expression (Fig. 1a), attenuated caspase-3 activity and HKM apoptosis (Figure S1). These findings were confirmed using CHOP-siRNA. Transfection with CHOP-siRNA down-regulated CHOP expression at mRNA (Fig. 1c) and protein level (Fig. 1a) besides attenuating *M. fortuitum*-induced HKM apoptosis (Figure S1). Our results for the first time implicated ER-stress induced CHOP in *M. fortuitum*-induced apoptosis and corroborate with earlier studies suggesting the pro-apoptotic role of CHOP in mycobacterial pathogenesis.

### Mobilization of (Ca^2+^)_ER_ into mitochondria led to mitochondrial dysfunction

**Fig. 2:**
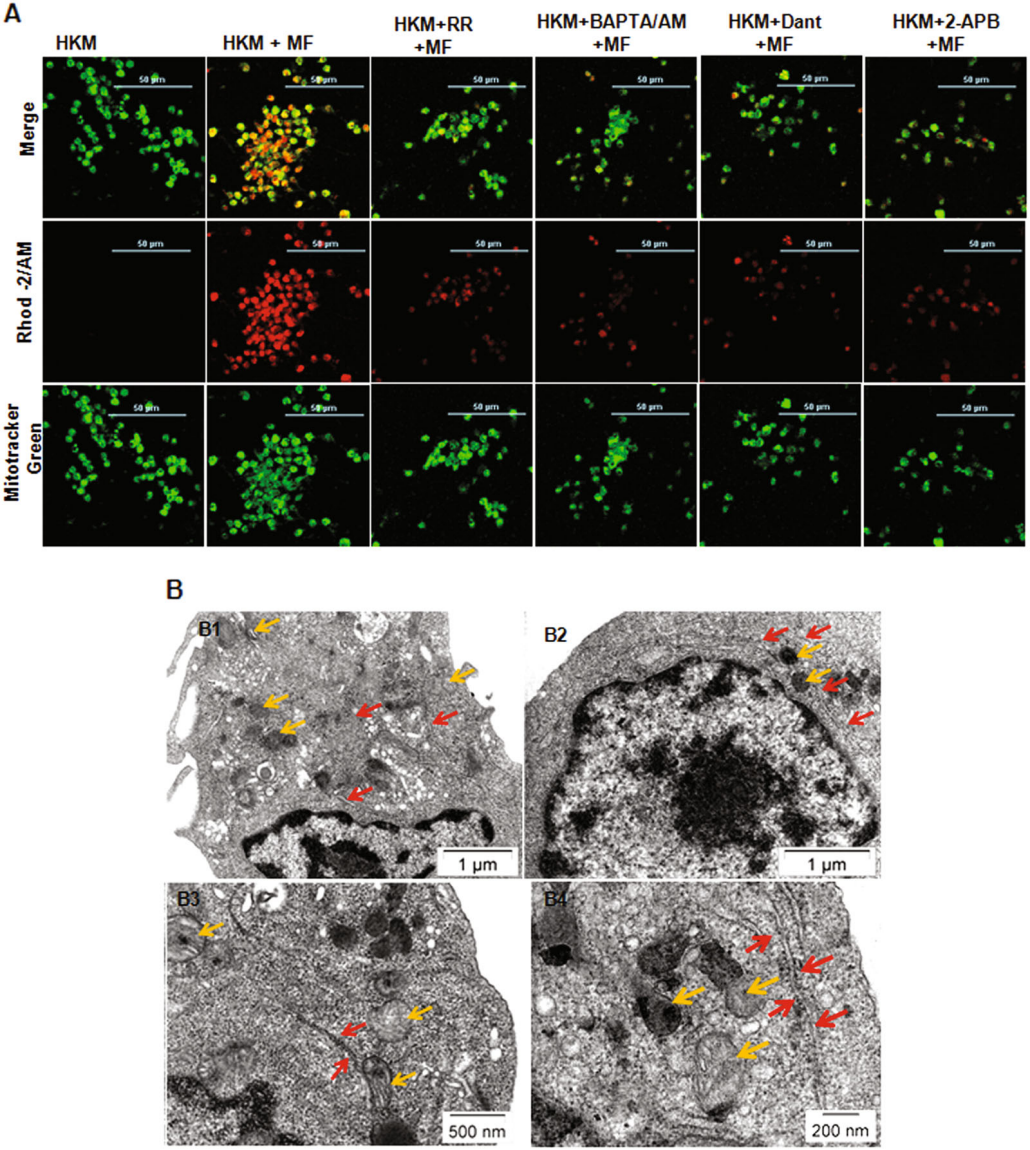
Close apposition of ER and mitochondria leads to mitochondrial-Ca^2+^ overload in *M. fortuitum* infected HKM. **a** HKM pre-treated with or without indicated inhibitors were infected with *M. fortuitum* and mitochondrial-Ca^2+^ uptake studied 6 h p.i. by Rhod-2/AM and Mitotracker green marker. The images are representative of three independent experiments and observed under confocal microscope ( × 40). **b** Transmission electron microscopy of uninfected HKM (B1), *M. fortuitum* infected HKM at 6 h p.i. (B2) and 24 h p.i. (B3, B4). The images are representative of three independent experiments. HKM, control headkidney macrophage; HKM + MF, HKM infected with *M. fortuitum*; HKM + RR + MF, HKM + BAPTA/AM + MF, HKM + Dant + MF, HKM + 2-APB + MF, HKM pre-treated with RR, BAPTA/AM, Dant and 2-APB respectively and infected with *M. fortuitum*. Yellow arrow,mitochondrion; Red arrow, ER

Mitochondrial dysfunction due to Ca^2+^ overload is keystone in determining the fate of mycobacteria-infected macrophages^26, 27^. HKM were stained with Rhod-2/AM and Mito-Tracker Green FM and observed under the confocal microscope. The increase in Rhod-2 AM fluroscence clearly indicates increased (Ca^2+^)_m_ uptake following 1 h of adding the bacteria with peak fluroscence recorded at 6 h p.i (Figure S2). Calcium influx to the mitochondria occurs through MUP^16^ and to explore this HKM were pre-treated with MUP blocker ruthenium red (RR) and (Ca^2+^)_m_ dynamics monitored at 6 h p.i. We observed Rhod-2/AM fluroscence intensity was reversed in presence of RR (Fig. 2a) suggesting (Ca^2+^)_m_ influx in *M. fortuitum*-infected HKM is uniporter driven.

To investigate the mobilization of (Ca^2+^)_ER_ to mitochondria HKM were pre-treated separately with Dant, 2-APB and (Ca^2+^)_m_ uptake monitored. The decrease in Rhod-2/AM fluorescence in presence of Dant and 2-APB (Fig. 2a), clearly proved the mobility of Ca^2+^ from ER to mitochondria. We reasoned, for the uptake of (Ca^2+^)_ER_ through MUP, the two organelles ought to come in close proximity thereby facilitating the process. *M. fortuitum*-infected HKM were examined by TEM at 6 h and 24 h p.i. (Fig. 2b) and we observed spatial change in the sub-cellular organization with mitochondria in close apposition with ER, which appeared more evident in the HKM collected at 24 h p.i. (Fig. 2B3,B4).

**Fig. 3:**
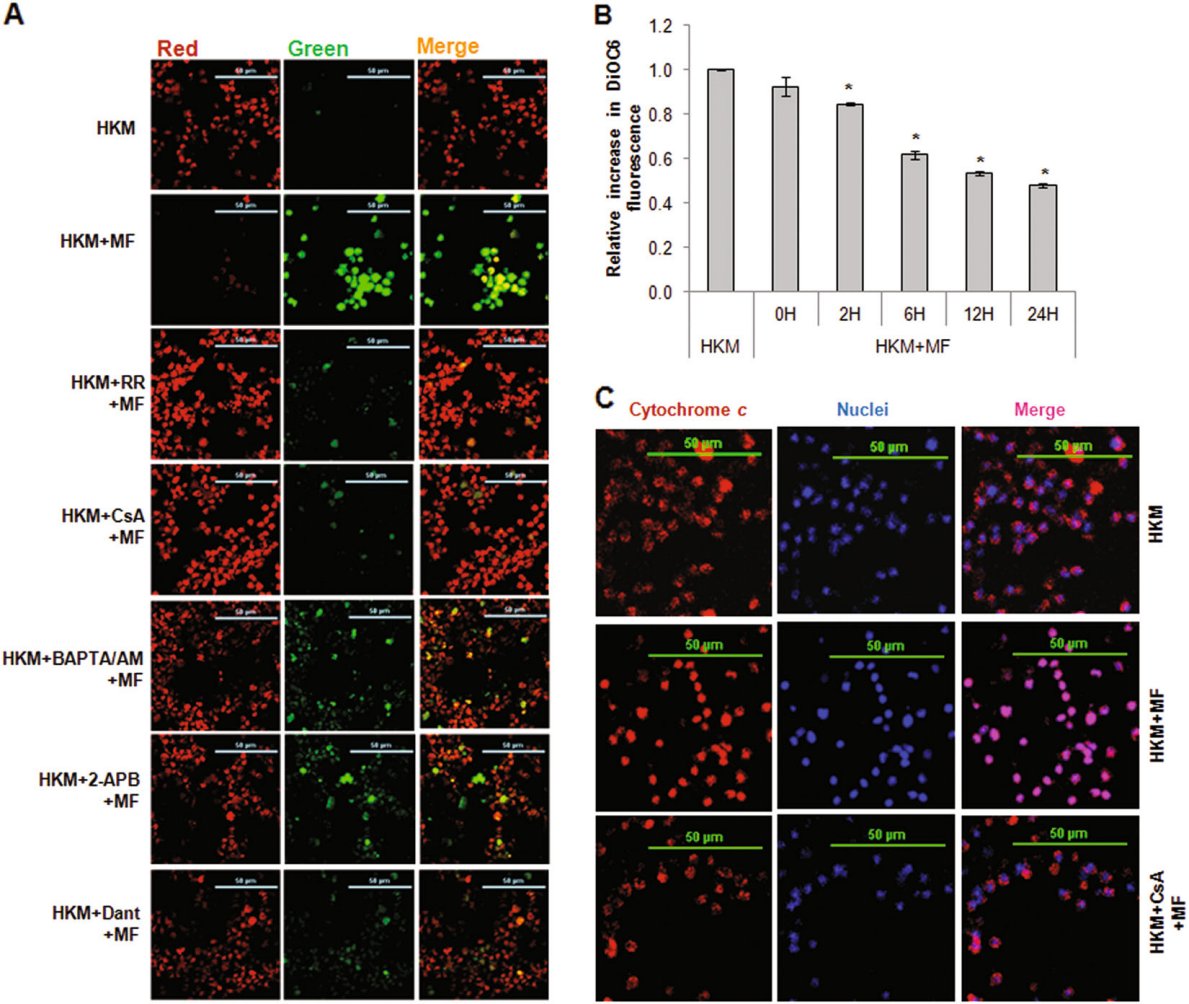
Alteration in cytosolic Ca^2+^ homeostasis induces mitochondrial dysfunction triggering *M. fortuitum*-induced HKM apoptosis. **a** The alteration in ΔΨm was studied using JC-1 dye. HKM pre-treated with or without indicated inhibitors were infected with *M. fortuitum* and at 12 h p.i. ΔΨm studied by confocal microscopy ( × 40). **b** MPT formation was studied using DiOC_6_ dye. HKM were infected with *M. fortuitum* for indicated time period and the relative fluorescence intensity of DiOC_6_ plotted. **c** HKM pre-treated with or without indicated inhibitors were infected with *M. fortuitum* and the cytosolic translocation of cytochrome *c* studied by immunoflurescence using TRITC- tagged secondary antibody at 24 h p.i. The nuclei were stained with DAPI. The images are representative of three independent experiments and observed under confocal microscope ( × 40). Vertical bars represent mean ± SE (*n* = 3).*P < 0.05, compared to HKM. HKM, control head kidney macrophage; HKM + MF, HKM infected with *M. fortuitum*; HKM + RR + MF, HKM + CsA + MF, HKM + BAPTA/AM + MF, HKM + Dant + MF, HKM + 2-APB + MF, HKM pre-treated with RR, CsA, BAPTA/AM, Dant, 2-APB respectively and infected with *M. fortuitum*

The elevation in (Ca^2+^)_m_ reduces mitochondrial membrane potential (ΔΨm)^28^. We monitored the changes in ΔΨm in *M. fortuitum*-infected HKM at different time points using the JC-1 dye. The increase in green fluorescence^29^ indicated time-dependent reduction in ΔΨm in infected HKM (Figure S3). Pre-treatment of HKM with specific inhibitors RR, Dant and 2-APB restored ΔΨm (Fig. 3a), which confirmed the uptake of (Ca^2+^)_ER_ on mitochondrial dysfunction. The loss in ΔΨm leads to the formation of MPT^28^. The cell-permeant, green-fluorescent, lipophilic dye DiOC_6_ accumulates in mitochondria and its release is a reliable indicator for ΔΨm loss and MPT pore opening^30^. When we compared DiOC_6_ fluoroscence levels in uninfected and *M. fortuitum*-infected HKM, significant loss in fluoroscence levels was noted in the infected HKM (Fig. 3b) which suggested *M. fortuitum* infection leads to loss in ΔΨm and MPT formation. Among several molecules released via MPT, pro-apoptotic cytochrome *c* is important. Hence, the next step was studying cytochrome *c* release in *M. fortuitum*-infected HKM. Our confocal microscopy images suggests the transloction of cytochrome *c* to the cytosol of *M. fortuitum*-infected HKM (Fig. 3c). Pre-treatment with MPT inhibitor CsA restored ΔΨm (Fig. 3a), retained DiOC_6_ (data not shown) and inhibited cytochrome *c* release (Fig. 3c) in infected HKM. To this we concluded that acquisition of (Ca^2+^)_ER_ impairs mitochondrial functioning triggering the apoptosis of *M. fortuitum*-infected HKM.

### Cytosolic Ca^2+^ imbalance activates the calpain/caspase-12 axis

**Fig. 4:**
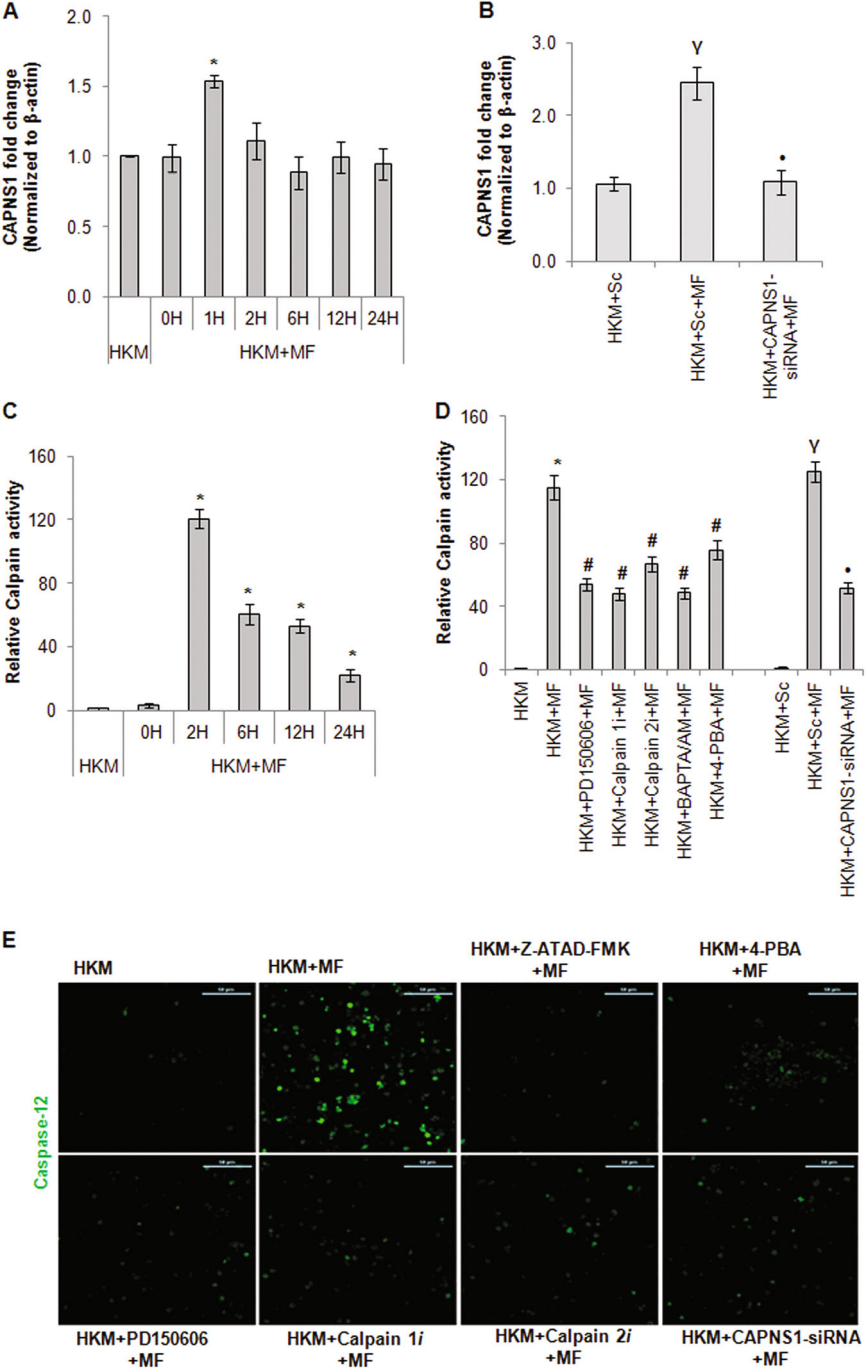
Activation of calpain/caspase-12 axis is consequent to ER-stress in *M. fortuitum* infected HKM. **a** HKM were infected with *M. fortuitum* and at indicated time p.i. CAPNS1-mRNA expression was quantified by qPCR. **b** HKM were transfected separately with CAPNS1-siRNA or scrambled siRNA and CAPNS1-mRNA expression was quantified by qPCR. **c** HKM were infected with *M. fortuitum* and at indicated time p.i. calpain activity was measured using assay kits. **d** HKM transfected separately with CAPNS1-siRNA, scrambled siRNA or pre-treated with indicated inhibitors were infected with *M. fortuitum* and calapin activity measured at 2 h p.i. **e** HKM pre-treated with indicated inhibitors or transfected with CAPNS1-siRNA were infected with *M. fortuitum* and caspase-12 activity was studied at 24 h p.i. using specific kit and observed under confocal microscope ( × 40). The images are representative of three independent experiments. Vertical bars represent mean ± SE (*n* = 3).*P < 0.05, compared to HKM; γ*P* < 0.05, compared to HKM + Sc; ^#^*P* < 0.05, compared to HKM + MF; •*P* < 0.05, compared to HKM + Sc + MF. HKM, control head kidney macrophage; HKM + Sc, HKM transfected with scrambled siRNA; HKM + MF, HKM infected with *M. fortuitum*; HKM + Sc + MF, HKM transfected with scrambled siRNA followed by *M. fortuitum* infection; HKM + CAPNS1-siRNA + MF, HKM transfected with CAPNS1-siRNA then infected with *M. fortuitum*; HKM + PD150606 + MF, HKM + Calpain1*i* + MF, HKM + Calpain2*i* + MF, HKM + 4-PBA + MF, HKM + BAPTA/AM + MF, HKM + Z-ATAD-FMK + MF, HKM pre-treated with PD150606, Calpain1*i*, Calpain2*i*, 4-PBA, BAPTA/AM, Z-ATAD-FMK respectively and infected with *M. fortuitum*

Calpains are implicated in apoptosis induced by several mycobacteria^11, 20^. In absence of earlier reports, we studied the role of calpain in *M. fortuitum*-induced HKM apoptosis. Realtime primers for the common 28-kDa regulatory subunit gene (CAPNS1) were designed and qPCR results demonstrated maximum CAPNS1-mRNA expression at 1 h p.i. (Fig. 4a). We followed this by measuring calpain activity using specific kit and noted maximum calpain activity at 2 h p.i. (Fig. 4c) and selected these two time points for subsequent studies.

Pre-treatment with BAPTA/AM attenuated calpain activity (Fig. 4d) indicating calpain activation to be Ca^2+^-dependent in *M. fortuitum-*infected HKM. Transfection studies were carried out with CAPNS1-siRNA and the results from RNAi studies demonstrated significant reduction in *M. fortuitum* induced calpain-mRNA expression (Fig. 4b), -protein activity (Fig. 4d) and HKM apoptosis (Figure S1). HKM were pre-treated with pan-calpain inhibitor PD150606 and the changes in calpain and caspase-3 activity and HKM apoptosis studied. We observed significant attenuation in calpain activity (Fig. 4d), caspase-3 activity and HKM apoptosis (Figure S1) in presence of PD150606. The inactive analog PD145305 had no effect on calpain and caspase-3 activation as well as HKM apoptosis (data not shown). Calpains exists in two isoforms and our interest was to identify their relative involvements in *M. fortuitum*-induced HKM apoptosis. In this direction, HKM pre-treated separately with calpain 1*i* and calpain 2*i* were infected with *M. fortuitum* and caspase-3 activation and apoptosis monitored. We observed calpain 1*i* and calpain 2*i* were equally effective in inhibiting calpain activation (Fig. 4d), caspase-3 activation and HKM apoptosis (Figure S1) suggesting both calpain isoforms contribute equivalently to *M. fortuitum*-pathogenesis.

ER-stress can induce the activation of calpains^31^. To correlate ER-stress with calpain activity the HKM were pre-treated with 4-PBA and calpain activity studied in the infected cells. We observed that 4-PBA pre-treatment led to significant reduction in calpain activiation (Fig. 4d) in *M. fortuitum*-infected HKM. These findings suggested ER-stress contribute towards pro-apoptotic calpain activation in *M. fortuitum* infected HKM.

We followed this by studying ER-stress induced caspase-12 activation. HKM were infected with *M. fortuitum* and caspase-12 expression monitored under the confocal microscope at 24 h p.i., the end point of the study. We observed significant caspase-12 activity in infected HKM. Pre-treatment with caspase-12 inhibitor Z-ATAD-FMK and 4-PBA inhibited caspase-12 activity (Fig. 4e) and attenuated *M. fortuitum*-induced HKM apoptosis (Figure S1). Thus, we concluded that ER-stress leads to pro-apoptotic caspase-12 activation in *M. fortuitum*-infected HKM.

Earlier studies suggested the involvement of calpains in caspase-12 activation^21^. To investigate this, the HKM were treated with PD150606, Calpain 1*i*, Calpain 2*i* or transfected with CAPNS1-siRNA prior to *M. fortuitum* infection and caspase-12 activity studied. We observed the down-regulation in caspase-12 activity in presence of PD150606, Calpain 1*i*, Calpain 2*i* and CAPNS1-siRNA respectively (Fig. 4e). PD145305 failed to inhibit caspase-12 activation (data not shown), suggesting the role of calpain on caspase-12 activation in *M. fortuitum-*infected HKM. Based on these findings we concluded that *M. fortuitum-*induced ER-stress lead to the activation of pro-apoptotic calpain/caspase-12 axis in HKM.

### Caspase-12 and cytochrome *c* instigate caspase-9 activation to expedite *M. fortuitum* induced-HKM apoptosis

**Fig. 5:**
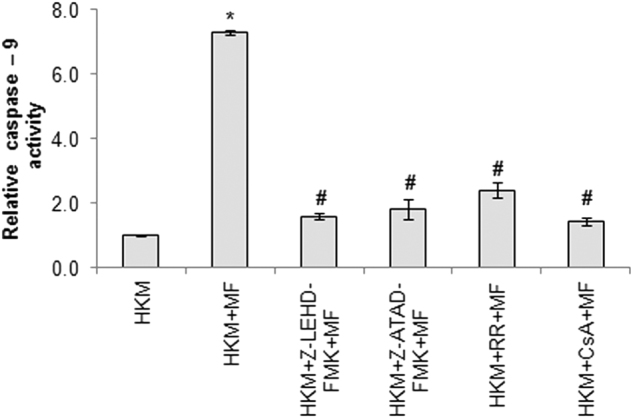
Cytochrome *c* and caspase-12 independently induce caspase-9 activation in *M. fortuitum* infected HKM. HKM pre-treated with or without indicated inhibitors were infected with *M. fortuitum* and caspase-9 activity measured 24 h p.i. Vertical bars represent mean ± SE (*n* = 3).*P < 0.05, compared to HKM; ^#^*P* < 0.05, compared to HKM + MF. HKM, control headkidney macrophage; HKM + MF, HKM infected with *M. fortuitum*; HKM + Z-LEHD-FMK + MF, HKM + Z-ATAD-FMK + MF, HKM + RR + MF, HKM + CsA + MF, HKM pre-treated with Z-LEHD-FMK, ZATAD-FMK, RR, CsA respectively and infected with *M. fortuitum*

The release of cytochrome c leads to activation of caspase-9 and cellular apoptosis^17^. We set out to determine the role of cytochrome *c-*caspase-9 axis in *M. fortuitum*-induced HKM apoptosis. Enhanced caspase-9 activity was noted in *M. fortuitum* infected HKM (Fig. 5) and pre-treatment with caspase-9 inhibitor Z-LEHD-FMK significantly attenuated caspase-3 activity and HKM apoptosis (Figure S1) implicating the involvement of caspase-9 in *M. fortuitum* pathogenesis. We extended our study and noted that caspase-9 activity was attenuated in the presence of MPT inhibitor CsA (Fig. 5) which ensured cytochrome *c* released due to MPT is critical for caspase-9 activation in *M. fortuitum-*infected HKM.

In an earlier study it was noted that caspase-12 influences caspase-9 further intensifying the apoptotic cascade^32^. To probe this, we pre-treated the HKM with the caspase-12 inhibitor Z-ATAD-FMK and assayed caspase-9 activity. It is evident from Fig. 5 that caspase-9 activity was attenuated in Z-ATAD-FMK pre-treated HKM. Based on these observations we propose that both cytochrome *c* and caspase-12 mediated pathways intersect at caspase-9 to expedite HKM death induced by *M. fortuitum*.

## Discussion

In the present study, we report that the cross-talk between ER and mitochondria aggravates down-stream apoptotic signaling in *M. fortuitum* infected HKM wherein, Ca^2+^ dynamics in the sub-cellular compartments plays a crucial role on expediting the death program.

The pro-apoptotic transcription factor CHOP is marker for ER-stress^7^. The expression of CHOP was significantly reduced in presence of BAPTA/AM, an intracellular Ca^2+^ chelator implicating alteration in intracellular Ca^2+^ homeostasis is closely related to ER-stress generation in *M. fortuitum* infected macrophages. We hypothesize, elevated cytoslic Ca^2+^ induces protein misfolding affecting protein loading in ER and BAPTA/AM might reduce misfolded proteins thus attenuating ER-stress induced by *M. fortuitum*. 4-PBA is low molecular weight chemical chaperone which has several biological effects of which inhibiting ER-stress is important^33^. It helps in stabilizing protein conformation thereby improving the folding capacity of ER and represses UPR^33, 34^. We hypothesized 4-PBA would prevent HKM apoptosis through inhibition of ER-stress induced CHOP expression. We observed that alleviating ER-stress with 4-PBA down-regulated CHOP expression coupled with decline in caspase-3 activity and HKM apoptosis. Besides, CHOP-siRNA suppressed caspae-3 activity and HKM apoptosis. CHOP has ‘versatile role’ in ER-stress mediated apoptosis. It down-regulates BCL-2 anti-apoptotic proteins at transcriptional level triggering the mitochondrial apoptotic cascade^35^. It has been also reported CHOP induces the transcription of ER oxidoreductin 1α (ERO1α) leading to hypoxic milieu in ER, that enhances downstream death signaling^35, 36^. In this context, it would be interesting to study the versatility of CHOP in *M. fortuitum* induced apoptosis.

Based on these observations, we propose that the ER-overload and unresolved ER-stress induced by *M. fortuitum* is a crucial trigger to induce HKM apoptosis. The role of cytosolic Ca^2+^ on ER-stress generation and macrophage apoptosis has been reported in several mycobacteria^10, 11^ and our results extends this to *M. fortuitum*, suggesting it to be common virulence trait for different mycobacterial species. This is the first report suggesting that *M. fortuitum* can induce ER-stress with pathological implications in host. Keeping the diverse host trophism of *M. fortuitum* in view it would be interesting to see whether same pathogenic mechanisms are employed by the bacteria to induce pathogenesis across species barrier.

Our results with 2-APB and Dant showed significant suppression in CHOP expression implicating the definite role of (Ca^2+^)_ER_ depletion in in *M. fortuitum* pathogenesis. The next step was to look for the likely down-stream targets induced by Ca^2+^ and calpain appeared attractive. Calpains are non-lysosomal cysteine proteases consisting of 80-kDa catalytic subunit and a common 28-kDa regulatory subunit, calpain small-1 (CAPNS1), encoded by CAPNS1 gene required for functioning^37, 38^. We observed over-expression of CAPNS1 mRNA and higher calpain activity and inhibiting the protease activity resulted in down regulation of caspase-3 and *M. fortuitum* induced HKM apoptosis. The presence of the two different tissue isoforms, calpain-1 and -2 is well documented in fish^39, 40^. Our results demonstrated that both isoforms are important for inducing *M. fortuitum* induced HKM apoptosis. Alleviating ER-stress with 4-PBA significantly reduced calpain activity suggesting calpain activation consequent to ER-stress generation in *M. fortuitum* infected HKM. Although the involvement of ER-calpain axis has been reported in the pathogensis of both atypical^11^ and typical mycobacteria^20^ our results constitute the first report in *M. fortuitum* suggesting calpain activation as an evolutionary conserved virulence attribute for mycobacteria.

We posited that the efflux of ER-Ca^2+^ into the cytoplasm through IP_3_ and RYR activates calpain to initiate downstream effects. Hence, calpain activity was studied in presence of 2-APB and Dant. Importantly, 2-APB and Dant, failed to completely abrogate calpain activity emphasizing Ca^2+^ efflux in *M. fortuitum* infected HKM involves multitude of pathways.

The activation of caspase-12 as a marker for ER-stress has recently been demonstrated in fish^41^. It has been observed that calpain cleaves the ER-resident pro-caspase-12 to active caspase-12 further intensifying the apoptotic cascade. Our results for the first time showed calpain-induced caspase-12 activation to be an important step in the pathogenesis of *M. fortuitum*. It was suggested that calpain-2 activation leads to cleavage of caspase-12^21^. We did not observe any difference in the ability of either calpain isoforms on caspase-12 activation suggesting caspase-12 to be substrate for both calpain-1 and -2 in *M. fortuitum* pathogenesis. We are currently studying the mechanisms of calpain dependency on caspase-12 activation in *M. fortuitum* pathogenesis.

An obligatory step in the ER-stress pathway is mitochondrial dysfunctioning with Ca^2+^ playing an active role on initiating the process^16, 17^. The participation of Ca^2+^ in *M. fortuitum* induced HKM apoptosis prompted us to explore ER-mitochondrial cross-talk in the pathogenesis induced by the bacterium. We observed overflow of (Ca^2+^)_ER_ to mitochondria of *M. fortuitum* infected HKM. Mitochondrial calcium overload alters mitochondrial membrane permeability and leads to opening of MPT. Our results showed that pre-treatment with MPT inhibitor CsA inhibited ΔΨm dissipation, caspase-3 activation and HKM apoptosis suggesting MPT formation to be associated with *M. fortuitum* pathogenesis. It has been reported that a close physical contact is pre-requisite for the mobility of Ca^2+^ from ER to mitochondria and MUP are the likely “hotspots” through which Ca^2+^ enters the mitochondria^6^. Ultra-structural studies depicted close apposition between the two organelles with mitochondria docked onto the ER. We used the specific MUP inhibitor RR and observed diminished mitochondrial Ca^2+^ uptake with concomitant decline in caspase-3 activity and HKM apoptosis. Similar reduction in mitochondrial Ca^2+^ load and MPT formation were also noted when the HKM were pre-treated with 2-APB and Dant. We propose that the interim association between the two organelles facilitates the efficient transfer of (Ca^2+^)_ER_ *via* MUPs leading to mitochondrial dysfunctioning and apoptosis of HKM.

MPT formation leads to overproduction of superoxide anions and release of pro-apoptotic cytochrome *c* into the cytosol. Increased amount of superoxide has been frequented with apoptosis induced by mycobacterial pathogens^10, 11^. Our preliminary results suggested the role of superoxide anions on *M. fortuitum* induced HKM apoptosis^24^. We hypothesize that MPT formation contributes to the overall process of apoptosis through the release of superoxide anions in the infected HKM. Blocking MPT formation by CsA significantly down-regulated cytochrome *c* release in cytosol suggesting close association between MPT formation and cytochrome *c* release in *M. fortuitum* infected HKM. We detected significant caspase-9 activity in *M. fortuitum* infected HKM which could be inhibited in presence of CsA suggesting MPT formation and cytochrome *c* release having a significant role on caspase-9 activation in *M. fortuitum* infected HKM. Earlier studies suggested a role of caspase-12 on activating the caspase-9/-3 axis^31, 42^. We observed, inhibiting caspase-12 significantly down-regulated the activation of caspase-9/3 axis, suggesting that caspase-9 can be activated by multiple pathways in *M. fortuitum* infected HKM. The involvement of caspase-9 is well documented in pathogenesis induced by several mycobacteria^12, 43–45^ our study extends this to *M. fortuitum*.

Mycobacteria-induced cell death depends on several factors including nature of bacterial strains, MOI, host cell types and durations of infection^46–48^. There are also reports suggesting mycobacteria causes caspase-dependent apoptotic and caspase-independent necrotic death depending on varied conditions of infection^48^. In this study, we observed caspase mediated apoptosis of catfish macrophages at 24 h p.i. and caspase-12/caspase-9 axis playing crucial role in triggering the process. The role of caspase-12 in ER-stress induced apoptosis is contentious with studies suggesting caspase-12 not to be part of UPR-induced apoptosis^49^. There are also reports that ER-stress induces caspase-independent necrosis^48^. In these studies, chemical stressers (tunicamycin, thapsigargin etc) have been used to induce and study the consequences of ER-stress, which may not be akin to pathogen induced stress. Nonetheless, presence of late apoptotic (AV^+^PI^+^) and necrotic (AV^−^PI^+^) subsets in *M. fortuitum* infected HKM along with inability of caspase inhibitors to completely abrogate cell death suggests a subset of HKM might be undergoing caspase-independent death. In this context, it would be interesting to study CHOP mediated necroptosis or necrosis in the immunopathogenesis of *M. fortuitum* at later time points or with higher MOI.

Cytochrome *c* also binds to IP_3_ receptors on the ER facilitating (Ca^2+^)_ER_ release^50^. We believe that besides its direct involvement in caspase-9 mediated apoptosis cytochrome *c* also contributes towards mitochondrial Ca^2+^ influx necessary for induction of HKM apoptosis. MPT formation leads to overproduction of superoxide anions and release of pro-apoptotic cytochrome *c* into the cytososl and increased amount of superoxide anions has been implicated in apoptosis induced by mycobacterial pathogens^10, 11^. We hypothesize that MPT formation also contributes to the overall process of apoptosis through the release of superoxide anion in the infected HKM.

The role of mitochondria in piscine mycobacteriosis is not clear. Recently it has been reported in *M. marinum*-zebrafish model that by modulating mitochondrial permeability transition pore formation mycobacteria induced programmed necrosis (necroptosis)^51^. We believe that these contradictions likely underline the complex and dynamic nature of the mycobacterial pathogenesis.

**Fig. 6:**
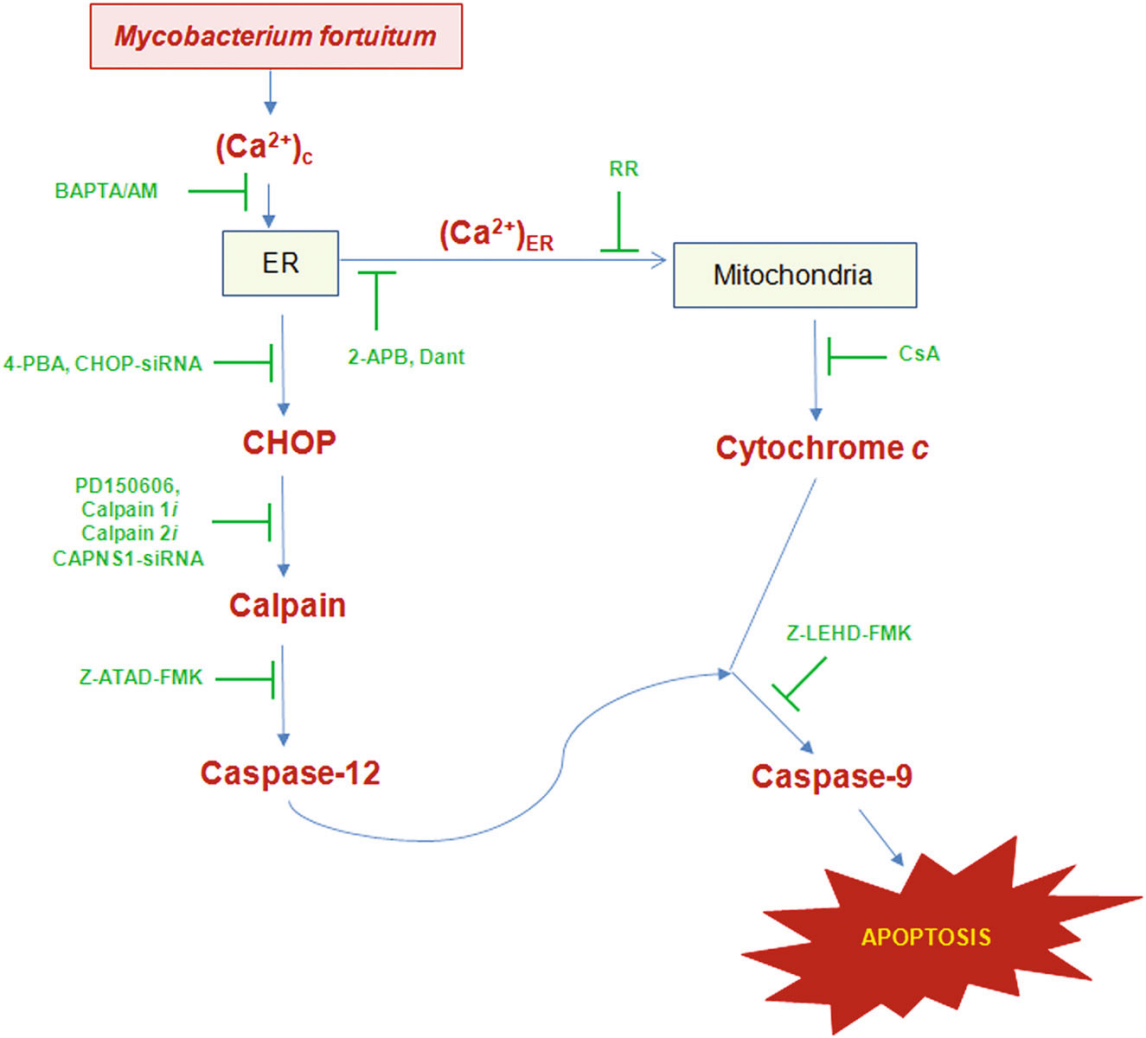
Overview of the work. *M. fortuitum*-induced alteration in Ca^2+^ homeostasis aggravates ER-stress. This leads to (i) activation of the calpain/ caspase-12 axis and (ii) mitochondrial dysfunction resulting in the cytosloic translocation of cytochrome *c*. The two pathways converge at caspase-9 leading to apoptosis of piscine macrophages

To conclude, our results established that Ca^2+^ dynamics of in sub-cellular compartments lead to ER-stress generation and mitochondrial dysfunctioning in *M. fortuitum*-infected HKM. We propose that altered cytosloic-Ca^2+^ triggers ER-stress accompanied with (Ca^2+^)_ER_ release. ER-Ca^2+,^ besides activating the calapin-caspase-12 axis also induces mitochondrial dysfunctioning; the two pathways converge at caspase-9 initiating caspase-3 mediated HKM apoptosis (Fig. 6). These findings would be useful for understanding the pathogenesis of *M. fortuitum* as well as controlling mycobacteriosis.

## Materials and methods

### Bacterial strains and growth conditions

*Mycobacterium fortuitum* (Strain MTCC 993) purchased from Microbial Type Culture Collection and Gene Bank (MTCC), Chandigarh, India were grown at 30 °C in standard Middlebrook 7H9 broth (HiMedia). The identity of the isolates was confirmed by AFB staining and 16 S rDNA sequencing. As the bacteria are sensitive to amikacin 50 µg/mL of the same was added to eradicate the extracellular bacteria^24^.

### Isolation of HKM and infection with *M. fortuitum*

All animal experiments were approved by the Animal Ethics Committee, University of Delhi (DU/ZOOL/IAEC-R/2013/34) and carried out in accordance with the protocols approved by The Prevention of Cruelty to Animals Act, Govt. of India. The methods for catfish (*Clarias* sp) maintenance and the protocols for obtaining HKM and infecting them with *M. fortuitum* (multiplicity of infection (MOI 10)) has been described earlier^24^.

### Inhibitors used

Intrcellular Ca^2+^ chelator (1, 2-Bis (2-aminophenoxy) ethane-N,N,N′,N′-tetraacetic acid tetrakis (acetoxymethyl ester), BAPTA/AM, 5 μM)), ER-stress alleviator (4-Phenyl butyric acid, 4-PBA, 10 µM), IP3 receptor antagonist (2-Aminoethyl diphenylborinate, 2-APB, 100 µM), calpain 1 inhibitor (N-acetyl-leucyl-leucyl-norleucinal, Calpain 1*i*, 50 µM), calpain 2 inhibitor (N-acetyl-leucyl-leucyl-methioninal, Calpain 2*i*, 50 µM), mitochondrial uniporter inhibitor (Ruthenium Red, RR, 20 µM), were purchased from Sigma. Pan-calpain inhibitor ([3-(4-iodophenyl)-2-mercapto-(Z)-2-propenoic acid], PD150606, 50 μΜ), negative control for calpain inhibitor (2-mercapto-3-phenypropionic acid, PD145305, 50 μM), rynodine receptor blocker (Dantrolene, Dant, 20 µM) were purchased from Calbiochem. MPTP blocker (Cyclosporin A, CsA, 5 µM) was from US Biological. Caspase-12 inhibitor (Z-ATAD-FMK, 10 μΜ) and caspase-9 inhibitor (Z-LEHD-FMK, 10 μΜ) were purchased from Biovision. Cytotoxicity test was done to determine the concentration of inhibitors used (data not shown). The inhibitors were added to the cell culture 1 h prior to the *M. fortuitum* infection and maintained throughout the experiment. The viability of HKM treated with the indicated concentrations of the inhibitors remained maintained at all-time points as checked by the trypan blue (0.4%) dye exclusion method. The concentrations of different inhibitors used for the study also had no effect on bacterial growth *per se* when added to Middlebrook 7H9 or complete-RPMI.

### siRNA Transfection

The siRNA transfection was carried out using HiPerFect Transfection Reagent (Qiagen), as described earlier^24, 52^. Transfection efficiency was confirmed by Real-Time PCR, protein and apoptosis assays. Five nano mole each of targeted CHOP [SENSE AUGAAGACUUGCAAGAUAUdTdT & ANTISENSE AUAUCUUGCAAGUCUUCAUdTdT], CAPNS1 [SENSE CAUGGACUUCGACAACUACdTdT & ANTISENSE GUAGUUGUCGAAGUCCAUGdTdT], and siRNA Universal negative CONTROL (Sigma) were used for this study.

### RNA isolation, cDNA synthesis, cloning, amplification, sequencing and quantative real-time PCR

HKM (2 × 10^7^) transfected separately with or without targeted or scrambled siRNA were infected with *M. fortuitum* and at indicated time p.i. the total RNA was isolated using TRIZOL (Sigma). cDNA was prepared from 1 μg of DNase treated (RNase-free) RNA using first strand cDNA synthesis kit as per manufacturer’s instructions (MBI Fermentas). Degenerate primers were designed using the homologous stretch across fish for CHOP and all vertebrates for the common calpain small sub-unit (CAPNS1) as the template (Table S1). The cDNA was amplified; the amplicons extracted using HiPura gel extraction kit (HiMedia), cloned into pGEM-T EASY vector (Promega) and sequenced (Macrogen). The sequences obtained (Table S2) were aligned to nBLAST and submitted to EMBL or NCBI database. The sequence for CHOP (accession number EMBL-LK054407) showed 80 % identity with CHOP-mRNA sequence of zebrafish (*Danio rerio*) and the sequence for CAPNS1 (accession number NCBI-KM242108) showed 80 % sequence identity with CAPNS1 sub-unit of Atlantic salmon (*Salmo salar*).

The quantification of CHOP and CAPNS1 mRNA were performed using SYBR green PCR Master Mix (Applied Biosystems) by Real-Time PCR (ABI ViiA, Applied Biosystems) as described earlier. The gene specific real-time primers for CHOP (FP:5′- GTTGGAGGCGTGGTATGAAG-3′; RP:5′-GAAACTCCGGCTCTTTCTCG-3′) and CAPNS1 (FP:5′-ACGGGAAAACTGGGGTTCG-3′; RP:5′-TGCTTATAGACAGCCTGCCAC-3′) have been used. Expression levels of target genes were analyzed by comparative ΔΔC_T_ method using β-actin as the internal control (endogenous control) and uninfected HKM (0 h) was used as the calibrator^24^.

### Apoptosis study

HKM (1 × 10^6^) transfected or pre-treated with or without indicated concentrations of targeted or scrambled siRNAs or specific inhibitors were infected with or without *M. fortuitum* (MOI 10) and apoptosis studied at 24 h p.i. by Hoechst 33342 (Sigma) and annexinV-FITC & propidium iodide (AV-PI, BD-Pharmingen) staining in fluroscence microscope ( × 40, Nikon Eclipse 400) as described earlier^24^.

### Immunofluorescence studies

HKM (5 × 10^6^) transfected or pre-treated with or without targeted or scrambled siRNAs and specific inhibitors were infected with or without *M. fortuitum*. At the indicated time p.i. the HKM were washed and fixed in methanol and were incubated in blocking and permeabilizing solution (PBS, 2 mg/mL BSA, 0.2 mg/mL saponin) for 1 h at room temperature. The cells were washed and incubated with primary antibodies; CHOP (mouse, 1:100, Cell Signalling Technology) and cytochrome *c* (mouse, 1:100, Biovision) separately overnight at 4 °C. The HKM were washed in PBST (PBS containing 0.1 % Tween-20) and stained with FITC or TRITC conjugated secondary antibodies (1: 250) for 3 h at 30 °C and visualized under confocal microscope ( × 40 oil immersion, 1.30 NA, Nikon Eclipse A1Rsi-T*i*E-300)^41^. Nuclei were stained with DAPI (1 μg/mL) before mounting on microslide.

### Imaging analysis of (Ca^2+^)_m_ uptake

The HKM (2 × 10^6^) pre-treated with or without specific inhibitors were infected with or without *M. fortuitum*. At indicated time p.i. the cells were washed, loaded simultaneously with Rhod-2/AM and mitotracker green (50 nM, Molecular Probes), incubated at 30 °C for 30 min then washed, mounted on microslide with cover slips using fluoroshield and visualized under confocal microscope ( × 40 oil immersion, 1.30 NA, Nikon Eclipse A1Rsi-T*i*E-300).

### Measurement of ΔΨm

The changes in ΔΨm and the induction of the mitochondrial permeability transition (MPT) were studied using JC1 (Cayman) and DiOC_6_ (Sigma) dyes respectively. In case of JC-1, cells with a high ΔΨm were those forming J-aggregates and in case of DiOC_6,_ high ΔΨm was attributed to cells with a high fluorescence signal.

HKM (2 × 10^6^) pre-treated with or without indicated concentrations of different inhibitors were infected with *M. fortuitum* for the indicated time p.i. and were loaded with DiOC_6_ (100 nM) during the last 30 min of infection then lysed in deionized water, and the reduction in the accumulation of DiOC_6_ was read in a fluorimeter (HT synergy) at excitation and emission wavelengths of 488 and 500 nm respectively. The relative change in fluorescence was plotted.

In parallel study, HKM (2 × 10^6^) pre-treated with or without indicated concentrations of different inhibitors were infected with or without *M. fortuitum* for indicated time periods. The cell pellet was harvested, washed with phosphate buffered saline and loaded with JC-1 (20 µM, Cayman) for 20 min. The cells were washed, mounted in microslide with cover slips using fluoroshield and the red/green fluorescence was digitized at indicated time p.i. using confocal microscope ( × 40 oil immersions, 1.30 NA, Nikon Eclipse A1Rsi-T*i*E-300).

### Transmission electron microscopy

HKM (2 × 10^7^) uninfected or infected with *M. fortuitum* for the indicated time period were washed and fixed with 2.5 % glutaraldehyde (Polaron, Biorad) in 0.1 M phosphate buffer (pH 7.4). The fixed HKM were processed as reported earlier^24^ and examined under Tecnai 12 Bio-twin transmission electron microscope (FEI, 80 kV).

### Calpain assay

Calpain activity was studied using a fluorogenic activity assay kit (Calbiochem). Briefly, HKM (1 × 10^5^) pre-treated with or without targeted or scrambled siRNAs and specific inhibitors were infected with or without *M. fortuitum*. The HKM were washed at indicated time p.i., lysed, calpain activity measured at 360 nm excitation and 460 nm emission respectively and the relative calpain activity plotted.

### Caspase assay (caspase-12, caspase-9 and caspase-3)

Caspase-12, caspase-9 and Caspase-3 were studied using specific assay kits according to the instructions of the manufacturer (Biovision) and using the reagents supplied with the kit. Briefly, HKM (1 × 10^6^) pre-treated with or without indicated concentrations of different inhibitors or targeted or scrambled siRNAs were infected with or without *M. fortuitum* for 24 h. The cells were washed and FITC-tagged caspase-12 inhibitor Z-ATAD-FMK was added into each culture and left for 30 mins at 30 °C followed by washing in fluorescein wash buffer. The HKM were mounted on microslides with cover slips using fluoroshield and analyzed under confocal microscope ( × 40 oil immersion)^41^.

For caspase-9 and caspase-3 assays the HKM were washed, re-suspended in 50 µl of lysis buffer and incubated on ice for 10 min. The cell lysate was collected by centrifugation at 10,000 × *g* for 5 min at 4 °C. To 50 μl of cell lysate, 50 μl of 2 × reaction buffer containing DTT (10 mM), PMSF (5 mM) and specific substrates were added, the mixture was incubated at 37 °C for 5 h and the absorbance read at 405 nm in a micro-plate reader (HT synergy)^40^.

### Statistical analysis

Mean ± SE were calculated for each parameter considered in the present study in the different groups of fish. Pair wise comparison was done by employing *t*-test: two samples using unequal variance to determine the statistical significance between the groups. The value of *p* < 0.05 was considered statistically significant.

## Electronic supplementary material

Supplementary Information
