# Supplementary material for: Mycobacterium fortuitum-induced ER-Mitochondrial calcium dynamics promotes calpain/caspase-12/caspase-9 mediated apoptosis in fish macrophages
Source: Cell Death Discov. 2018 Feb 20;4:30. doi: 10.1038/s41420-018-0034-9 (PMC5841318; doi:10.1038/s41420-018-0034-9)
Supplement: Supplementary file 1 — Supplementary Information [file 41420_2018_34_MOESM1_ESM.docx]

**Supplementary information**

**
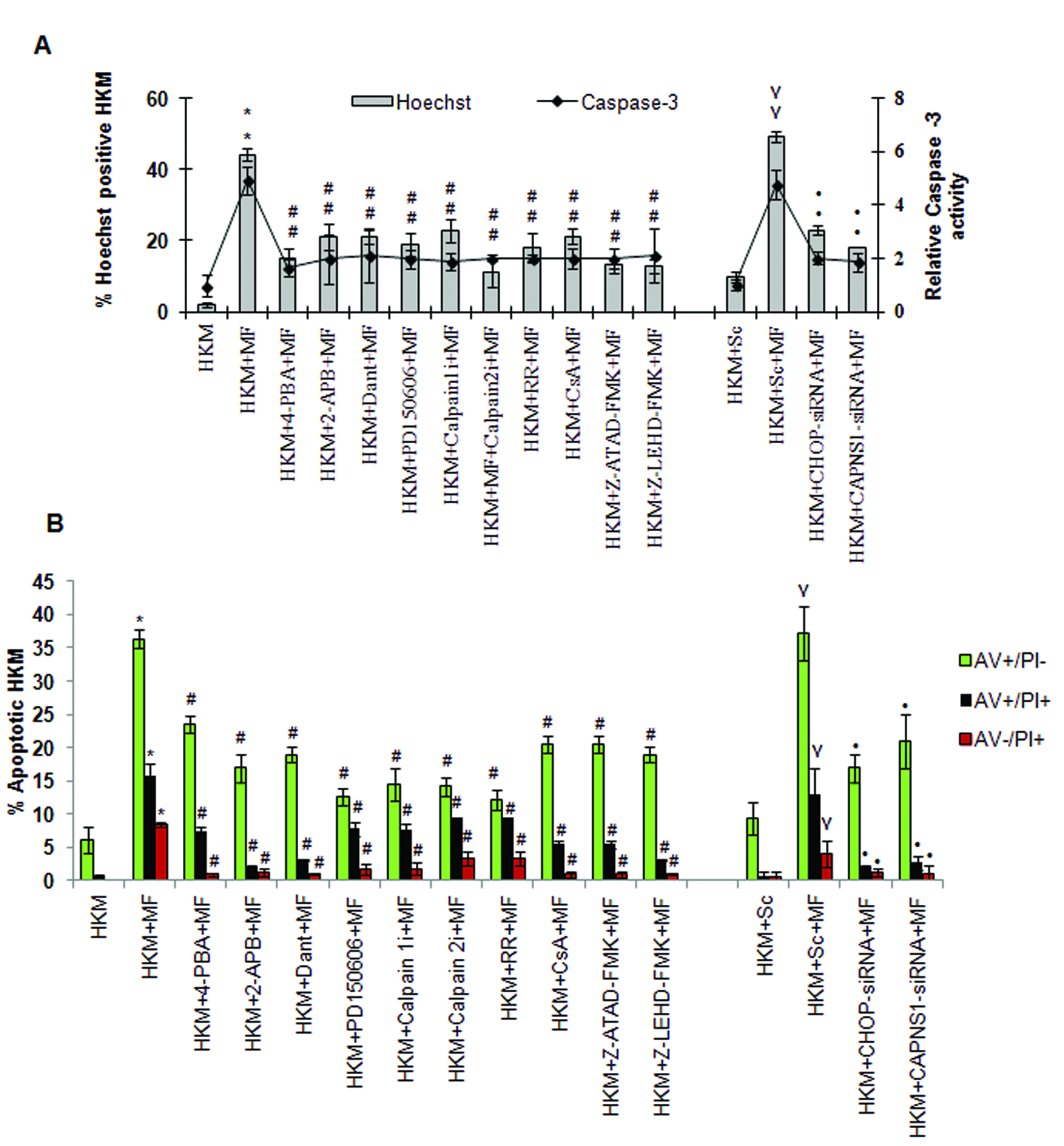
**

**Figure S1. *M. fortuitum* induces HKM apoptosis**

HKM were pre-treated separately with indicated inhibitors or transfected with specific siRNAs prior to *M. fortuitum* infection and at 24 h p.i. apoptosis measured by (A) Hoechst 33342 staining and relative caspase-3 activity and (B) AV/PI staining. Vertical bars represent mean ± SE (n=3).**P*<0.05, compared to HKM; **^γ^***P*<0.05, compared to HKM+Sc; ^#^*P*<0.05, compared to HKM+MF; **^•^***P*<0.05, compared to HKM+MF+Sc. HKM, control headkidney macrophage; HKM+Sc, HKM transfected with scrambled siRNA; HKM+MF, HKM infected with *M. fortuitum*; HKM+Sc+MF, HKM transfected with scrambled siRNA followed by *M. fortuitum* infection; HKM+CHOP-siRNA+MF, HKM transfected with CHOP-siRNA followed by *M. fortuitum* infection; HKM+CAPNS1-siRNA+MF, HKM transfected with CAPNS1-siRNA followed by *M. fortuitum* infection; HKM+4-PBA+MF, HKM+2-APB+MF, HKM+Dant+MF, HKM+PD150606+MF, HKM+Calpain1*i*+MF, HKM+Calpain2*i*+MF, HKM+RR+MF, HKM+CsA+MF, HKM+Z-ATAD-FMK+MF, HKM+Z-LEHD-FMK+MF, HKM pre-treated with 4-PBA, 2-APB, Dant, PD150606, Calpain 1*i*, Calpain 2*i*, RR, CsA, Z-ATAD-FMK or Z- LEHD-FMK respectively and infected with *M. fortuitum*.

**
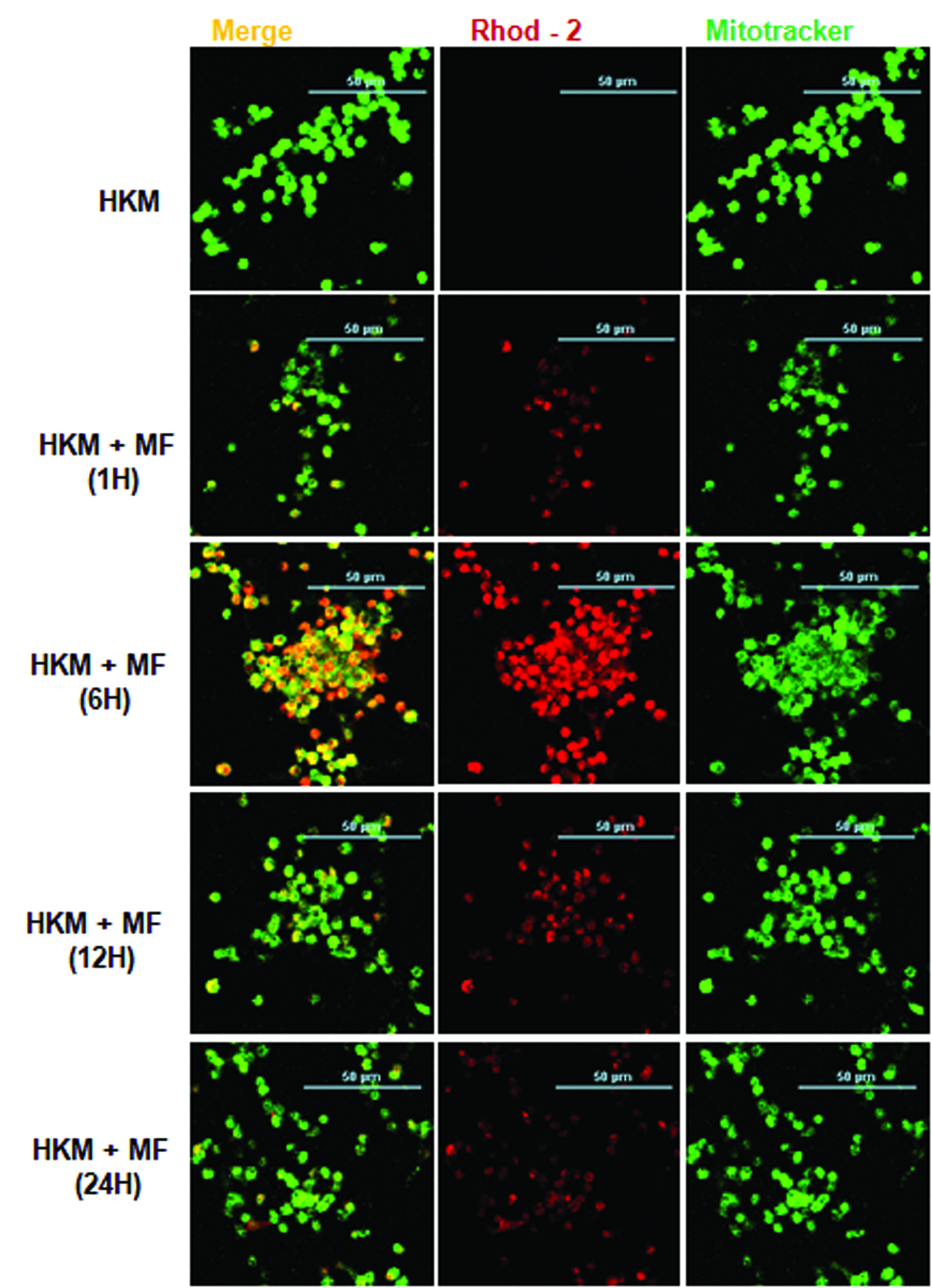
**

**Figure S2. *M. fortuitum* infection lead to mitochondrial Ca^2+^ mobilization**

HKM (uninfected or infected) were stained with Rhod-2/AM and mitotracker green marker and the mitochondrial-Ca^2+^ uptake studied at indicated time p.i. by confocal microscope. The images are representative of three independent experiments (×40). HKM, control headkidney macrophage; HKM+MF, HKM infected with *M. fortuitum*.


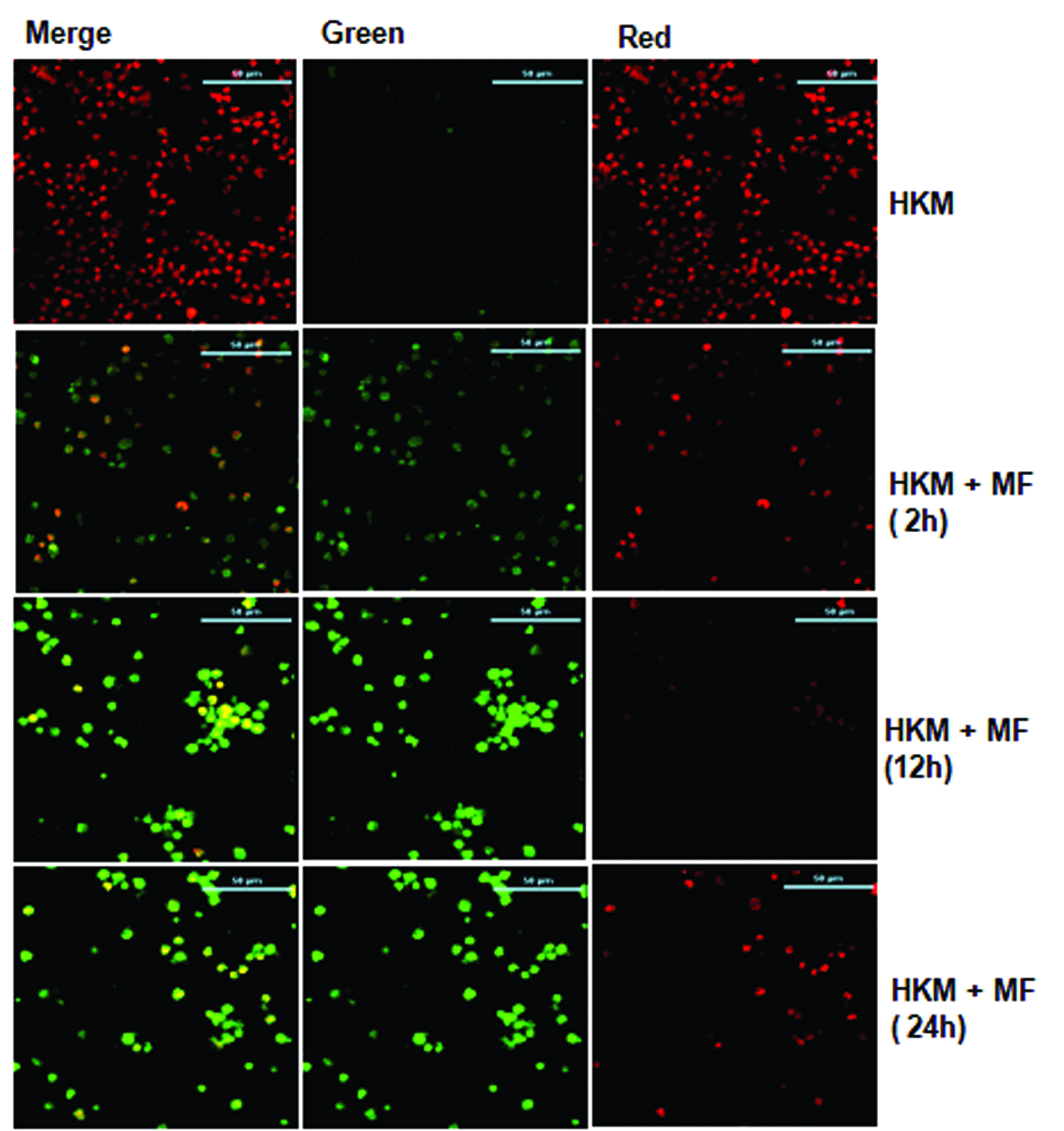


**Figure S3. *M. fortuitum* infection induces ΔΨm loss**

HKM infected with *M. fortuitum* for indicated time period were stained with JC-1 dye and the alteration of ΔΨm studied by confocal microscope. The images are representative of three independent experiments (×40). HKM, control headkidney macrophage; HKM+MF, HKM infected with *M. fortuitum*.

**Table S1. List of degenerate primers**

| **CHOP** | |
| --- | --- |
| **Forward** | **Reverse** |
| 5'-GGG CCG CTG TGT GGT GCA GAG-3‘  5' - TAC CCC CCT GGY GTG GGG CC -3'  5’ - GGT GCA GAG TTG GAG GCG TGG -3’ | 5'-CCC TTC GGT CAA CCA GGT GAG-3'  5’- YTC GAA RAA CTC GGG MGG MAG - 3’  5’- YTT CCT YTC GTT CTC CTG YTC- 3’ |
| **CAPNS1** | |
| 5' - CAG CTS GCT GGR GAY GAC ATG - 3‘  5' - GAT CTG AAR ACW GAT GGY TT - 3' | 5' - RTT RTC RAA ATC CAT GTT YCC - 3‘  5' - GMA CAT GGC RTC CAG YCT GAC - 3' |
| R = A/G; Y = C/T; K = G/T, M = A/C | |

**Table S2. Gene sequences**

| **Gene Name** | **Nucleotide sequence** |
| --- | --- |
| **CHOP**  (C/EBP  homologous  protein)/ DDIT3  (DNA-damage  inducible  transcript3)  Accession no.  **EMBLLK054407** | GGTGCAGAGTTGGAGGCGTGGTATGAAGACTTGCAAGATATATTGGGATCCGATGCAGGTGGGGCCAAGCACACACGCCCCCCACCCTGCGCCGAGAAAGAGCCGGAGTTTCTGGATGTCTTGGAGAGCTGCTCACTC |
| **CAPNS1**  (Calpain  small subunit 1)  Accession no.  **NCBIKM242108** | CAGCTCGCTGGGGATGACATGGAGGTGAGCCCCAATGAGCTGATGAGCATCCTCAACAAAATCATCTCTAAGCATGCCGACCTGAAGACTGATGGTTTTACAATCGAGTCCTGCGGGAGTATGGTGGCCGTCATGGACAGCGACAGCACGGGAAAACTGGGGTTCGAAGAGTTCAAGTACCTCTGGAACAACATCAAGAAGTGGCAGGCTGTCTATAAGCAGTATGATGCCGATCACTCCGGCGTGATCGGAGCGGATGAGCTGCCCAACGCGTTCCGAGCTGCAGGCTTCCCCCTGAGCGATCAGGTCTTCCAGCTTATCATCCGCAGGTACAGCGACGATAACGGCAACATGGACTTCGACAACTACATCGGATGCCTGGTCAGACTGGACGCCATGTG |
